# Supplementary material for: Effect of Polydopamine Coating of Cellulose Nanocrystals on Performance of PCL/PLA Bio-Nanocomposites
Source: Materials (Basel). 2023 Jan 27;16(3):1087. doi: 10.3390/ma16031087 (PMC9920865; doi:10.3390/ma16031087)
Supplement: Supplementary file 1 [file materials-16-01087-s001.zip › materials-2156701-supplementary.pdf]

Supplementary Materials

Table S1. Composition of PLA/PCL blends and nanocomposites in % wt.

PLA/PCL	Code	PLA	PLA_{dmf}	PLA/CNC	PLA/CNCd	PCL	PCL_{dmf}	PCL/CNC	PCL/CNCd
80/20	PLA_{dmf}	60	20			20			
	PCL_{dmf}	80					20		
	PLA/CNC	60		20		20			
	PCL/CNC	80						20	
	PLA/CNCd	60			20	20			
	PCL/CNCd	80							20
60/40	PLA_{dmf}	40	20			40			
	PCL_{dmf}	60				20	20		
	PLA/CNC	40		20		40			
	PCL/CNC	60				20	20		
	PLA/CNCd	40			20	40			
	PCL/CNCd	60				20			20
40/60	PLA_{dmf}	20	20			60			
	PCL_{dmf}	40				40	20		
	PLA/CNC	20		20		60			
	PCL/CNC	40				40		20	
	PLA/CNCd	20			20	60			
	PCL/CNCd	40				40			20
20/80	PLA_{dmf}		20			80			
	PCL_{dmf}	20				60	20		
	PLA/CNC			20		80			
	PCL/CNC	20				60		20	
	PLA/CNCd				20	80			
	PCL/CNCd	20				60			20

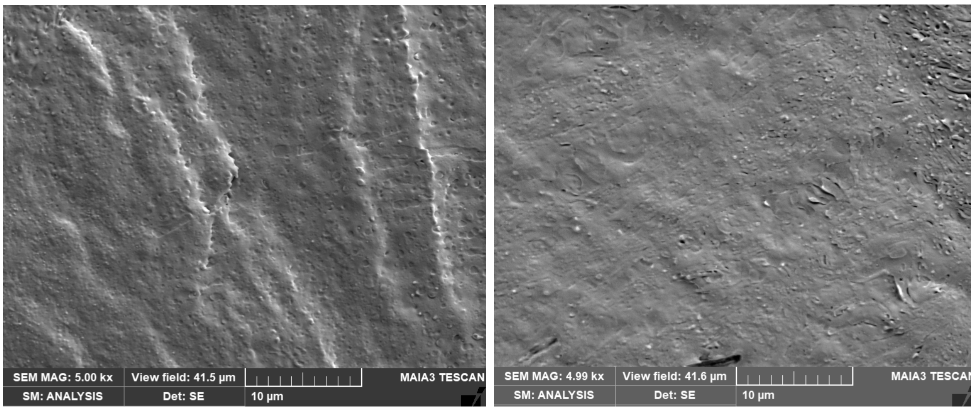

(a) (b)

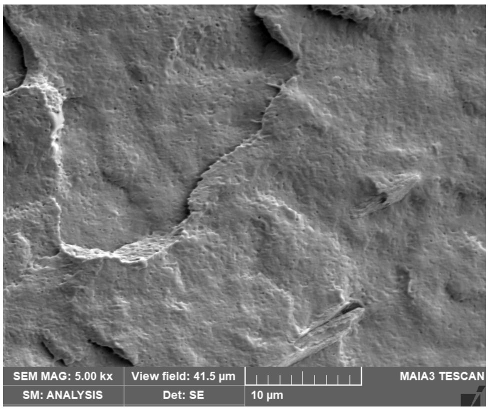

(c)

Figure S1. SEM images of specimens broken under liquid nitrogen (**a**) 20/80 PLA/CNC masterbatch (**b**) 40/60 PCL/CNC masterbatch, (**c**) 60/40 PLA/CNC masterbatch.

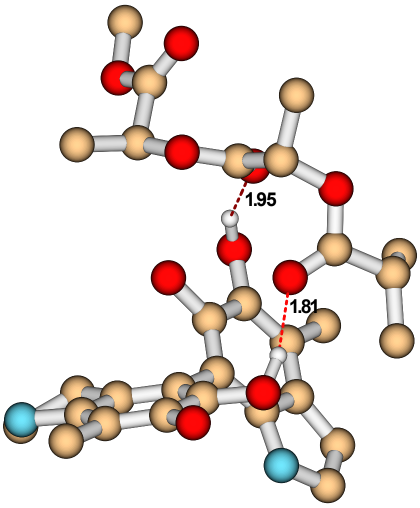

(a) Stabilization energy: 22.0 kcal/mol

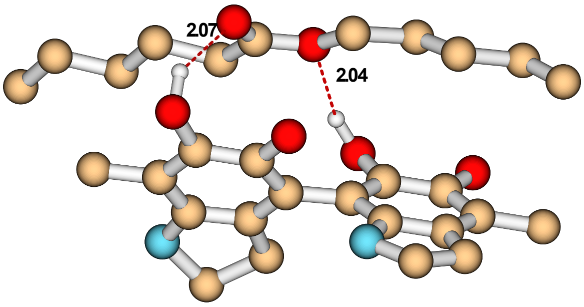

(b) Stabilization energy: 27.6 kcal/mol

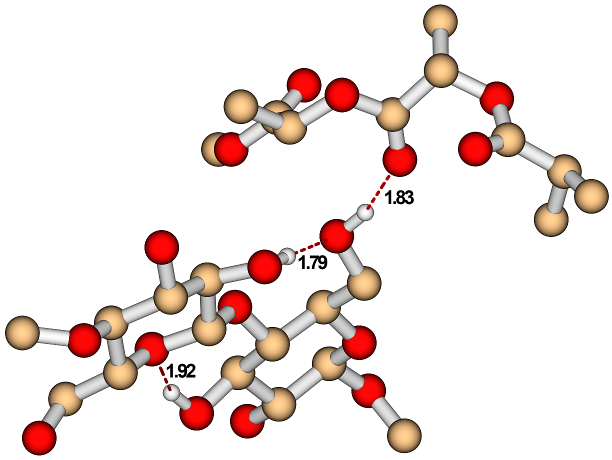

(c) Stabilization energy: 16.2 kcal/mol

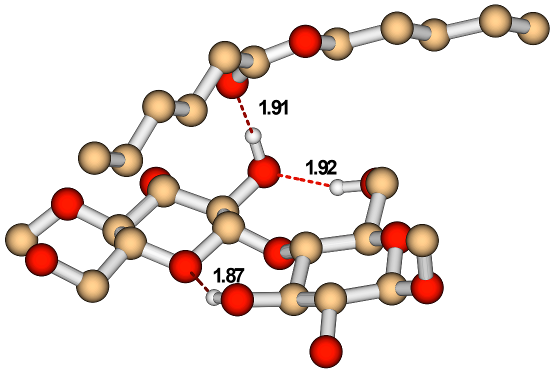

(d) Stabilization energy: 16.7 kcal/mol

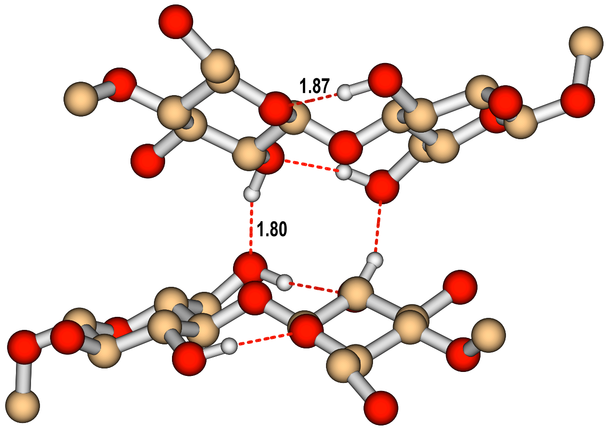

(e) Stabilization energy: 29.7 kcal/mol

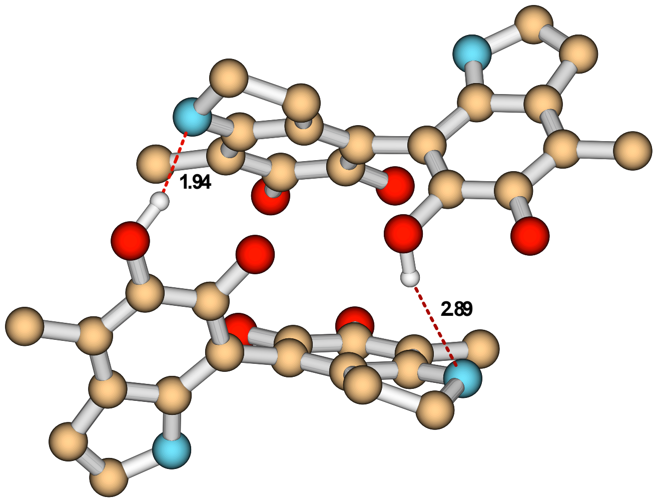

(f) Stabilization energy: 33.1 kcal/mol

Figure S2. (**a**) DFT model calculations of interactions between the structural units of PDA and PDL. Hydrogen atoms are omitted for clarity. **(b)** DFT model calculations of interactions between the structural units of PDA and PCL. Hydrogen atoms are omitted for clarity. **(c)** DFT model calculations of interactions between the structural units of CNC and PLA. Hydrogen atoms are omitted for clarity. **(d)** DFT model calculations of interactions between the structural units of CNC and PCL. Hydrogen atoms are omitted for clarity. **(e)** DFT model calculations of interactions between the structural units of CNC and CNC. Hydrogen atoms are omitted for clarity. **(f)** DFT model calculations of interactions between the structural units of PDA and PDA. Hydrogen atoms are omitted for clarity.

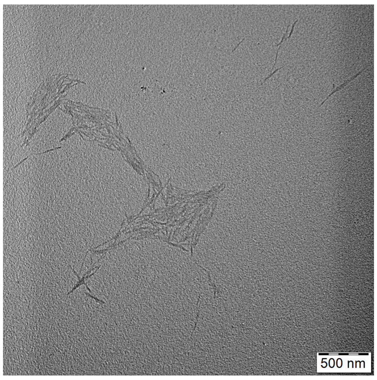

(a)

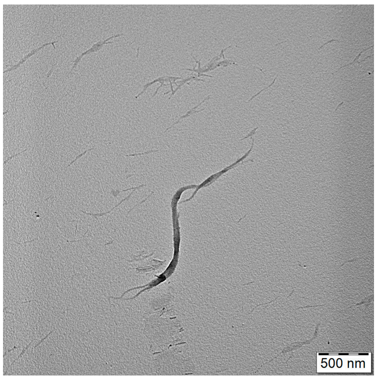

(b)

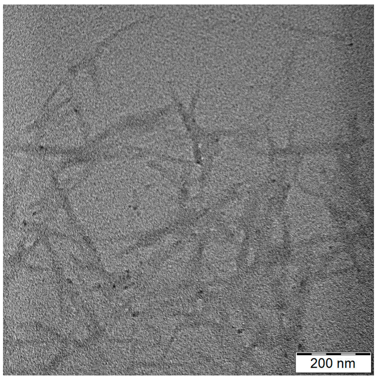

(c)

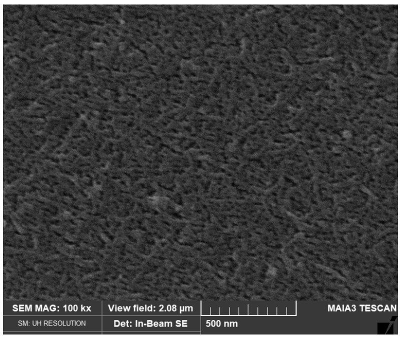

(d)

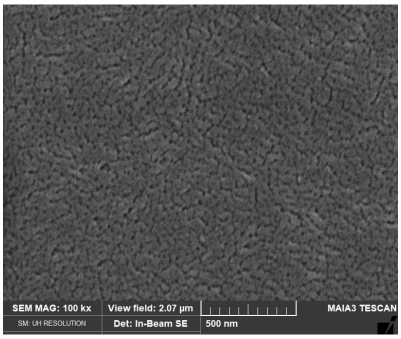

(e)

Figure S3. TEM images of (CNC) (**a**) and polydopamine-coated cellulose nanocrystals CNCd (**b,c**) and SEM images of (**d**) CNC and (**e**) CNCd confirming higher potential of Polydopanine-coated CNC for ordering

Rheological characterization of polymer components

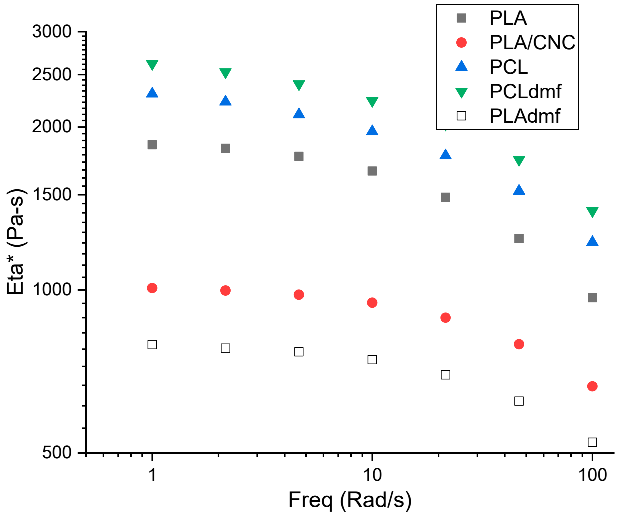

Figure S4. Viscosity of polymer components: DMF treatment of PLA and PCL identical to masterbatch preparation

From Figure S4 follows that viscosity of melt processed PLA and PCL are comparable, surprisingly, DMF treatment causes slight increase PCL viscosity but relatively marked decrease for PLA.

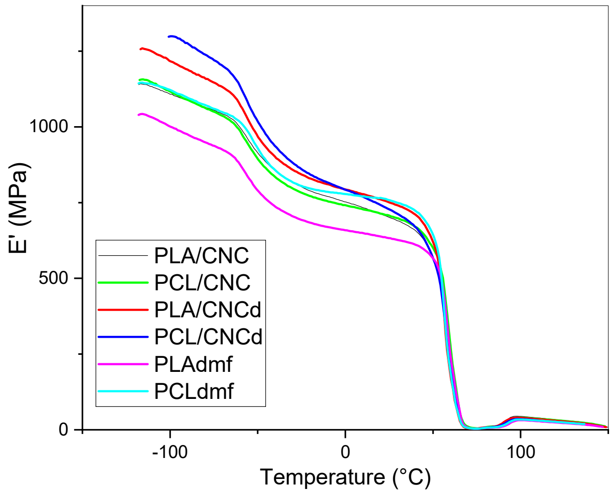

(a)

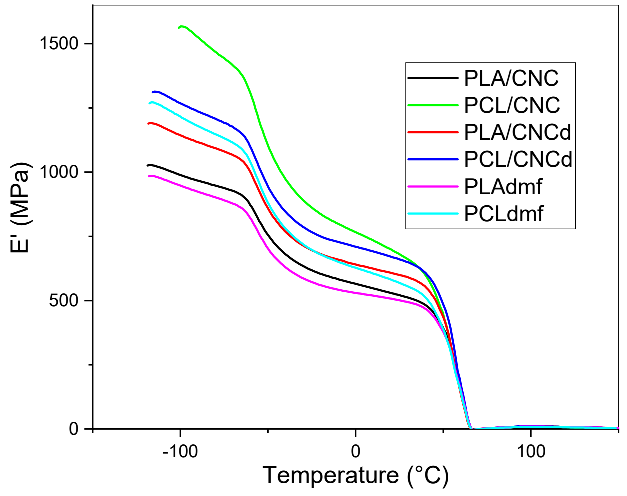

(b)

Figure S5. Temperature dependence of storage modulus of **a**) 60/40 PCL/PLA ratio; **b**) 40/60 ratio.

Table S2. Crystallinity of PCL, „total" crystallinity of PLA (initial + cold crystallization) + initial crystallinty of PLA.

**1st run**		**80/20**			**60/40**			**40/60**			**20/80**	
	**PCL [%]**	**PLAm [%]**	**PLAi [%]**	**PCL [%]**	**PLAm [%]**	**PLAi [%]**	**PCL [%]**	**PLAm [%]**	**PLAi [%]**	**PCL [%]**	**PLAi [%]**	**PLAm [%]**
PLA/CNC	71.57	30.17	3.25	59.76	31.42	4.02	57.02	30.12	3.51	54.99	28.06	4.77
PCL/CNC	70.65	27.73	3.03	61.14	29.10	3.86	57.49	29.18	4.71	59.68	29.45	8.51
PLA/CNCd	72.19	29.86	3.72	64.58	28.74	3.86	59.57	26.93	3.76	55.70	27.92	2.95
PCL/CNCd	70.52	27.76	3.15	58.35	25.81	2.98	55.95	27.52	2.62	53.15	26.67	4.47
PLAdmf	60.85	28.28	4.86	58.36	27.45	4.39	57.69	30.12	6.29	54.60	32.87	7.02
PCLdmf	60.90	25.56	4.13	55.61	26.84	3.29	56.60	27.71	6.16	53.27	27.42	8.64
